# Seeing red; the development of pON.mCherry, a broad-host range constitutive expression plasmid for Gram-negative bacteria

**DOI:** 10.1371/journal.pone.0173116

**Published:** 2017-03-03

**Authors:** Michael J. Gebhardt, Rachael K. Jacobson, Howard A. Shuman

**Affiliations:** Department of Microbiology, University of Chicago, Chicago, Illinois, United States of America; University of São Paulo FMRP/USP, BRAZIL

## Abstract

The development of plasmid-mediated gene expression control in bacteria revolutionized the field of bacteriology. Many of these expression control systems rely on the addition of small molecules, generally metabolites or non-metabolized analogs thereof, to the growth medium to induce expression of the genes of interest. The paradigmatic example of an expression control system is the *lac* system from *Escherichia coli*, which typically relies on the P*tac* promoter and the Lac repressor, LacI. In many cases, however, constitutive gene expression is desired, and other experimental approaches require the coordinated control of multiple genes. While multiple systems have been developed for use in *E*. *coli* and its close relatives, the utility and/or functionality of these tools does not always translate to other species. For example, for the Gram-negative pathogen, *Legionella pneumophila*, a causative agent of Legionnaires’ Disease, the aforementioned P*tac* system represents the only well-established expression control system. In order to enhance the tools available to study bacterial gene expression in *L*. *pneumophila*, we developed a plasmid, pON.mCherry, which confers constitutive gene expression from a mutagenized LacI binding site. We demonstrate that pON.mCherry neither interferes with other plasmids harboring an intact LacI-P*tac* expression system nor alters the growth of *Legionella* species during intracellular growth. Furthermore, the broad-host range plasmid backbone of pON.mCherry allows constitutive gene expression in a wide variety of Gram-negative bacterial species, making pON.mCherry a useful tool for the greater research community.

## Introduction

The *lac* operon promoter has been widely employed as a means for gene induction in a wide array of bacteria, ranging from Gram-negative to Gram-positive organisms. In many cases, however, the ability to control the expression of multiple genes within a given bacterial culture simultaneously is also desired. Examples of such experiments include fluorescent microscopy imaging techniques or assessing how two proteins function together and/or independently within a given bacterial cell. In these experiments, the ability to concurrently control the expression of multiple genes can greatly assist the researcher in studying a particular phenomenon. Not surprisingly, there have been a variety of additional induction systems developed to help solve this problem, including plasmid systems that are controlled by small molecules such as arabinose, tetracycline and xylose [1–3]. However, in many species, including *Legionella pneumophila*, alternative induction methods have either not been developed or are non-functional. In other experimental approaches, it is desirable to achieve constitutive gene expression in the absence of an inducing substance, if, for example the inducer is known to induce off target effects—such as inducing the expression of genes within the host bacterium, as could be the case for both IPTG and arabinose [1, 4].

In terms of gene expression control, the *lac* operon represents one of the most widely studied operons and is also one of the most utilized expression control systems for controlling gene expression in bacteria [5]. In the wild type *lac* operon, the lac repressor, encoded by the *lacI* gene, binds to the *lac* operator sequence, a 17 base pair segment of DNA with partial symmetry, to inhibit transcription of the *lacZYA* structural genes. The *lac* operon contains three operator sequences, O_1_, O_2_, and O_3_, and the Lac Repressor, which exists as a tetramer, is able to bind two of the three operator sites at any given time, to exert repression of *lacZYA* gene expression. The ability of the repressor to concurrently bind multiple operator sites allows for DNA looping, which further inhibits transcription initiation from the *lac* promoter [4]. However, many of the plasmids utilizing the *lac* system as a means to control gene expression often only include a single operator sequence to regulate gene expression, resulting in a decreased level of repression [1].

We report here the construction of pON.mCherry, a broad-host range vector, which results in constitutive expression of the widely used fluorescent protein, mCherry. Plasmid pON.mCherry is derived from pMMB207C, a derivative of the broad-host range plasmid RSF1010 [6]. The pON.mCherry plasmid thus replicates in a wide range of Gram-negative bacterial species and relies upon the widely used P*tac* promoter, a fusion between the *trp* and *lac* promoters, to drive constitutive gene expression [7].

## Materials and methods

### Strains and growth conditions

Bacterial strains are listed in Table 1. Except for *Legionella* spp., bacterial strains were routinely cultured in LB broth and/or agar plates at 37°C. *Legionella* spp. were cultured at 37°C on ACES-Buffered Charcoal Yeast Extract (CYE) plates or in ACES-buffered Yeast Extract (AYE) broth. Chloramphenicol was added when necessary at 30 μg/ml for solid medium and 20 μg/mL for axenic growth, except for *Legionella* and *Vibrio* spp., for which 5 μg/ml was added, and *Klebsiella*, which was cultured in the presence of 50 μg/ml chloramphenicol. When necessary, gentamicin was added to the growth media at a concentration of 15 μg/mL. *Acanthamoeba castellanii* (ATCC 30234) trophozoites were maintained in Peptone-Yeast extract-Glucose (PYG) medium at 30°C, as recommended by the ATCC. *Dictyostelium discoideum* AX4 trophozoites were grown axenically in HL5 medium with shaking at 125 rpm at room temperature.

**Table 1:** Strains and plasmids used in this study.

Strain or Plasmid	Relevant Properties	Source or Reference
Bacterial Strains		
*L*. *pneumophila* str. KS79	Wild type *Legionella pneumophila*	Lab Stock
*L*. *longbeachae*	ATCC 33462	ATCC
*L*. *gratiana*	ATCC 49413	ATCC
*L*. *parisiensis*	PF-209-C-C2	[8]
*Acinetobacter baylyi* str. ADP1	ATCC 33305	ATCC
*E*. *coli* DH5α		Lab Stock
*Salmonella typhimurium* str. LT2		Lab Stock
*Vibrio cholerae* str. C6707		Lab Stock
*Klebsiella aerogenes*		Lab Stock
Plasmids		
pMMB207c	pMMB207 *mobA*	[6]
pXDC31	pMMB207c derivative encoding GFP under control of Ptac, Chlor^R^	X. Charpentier
pXDC50	pMMB207c derivative encoding mCherry under control of Ptac, Chlor^R^	[9]
pXDC92	pBBR-derivative encoding mCherry under control of Ptac, confers kanamycin resistance	X. Charpentier
pON.mCherry	pXDC50 containing a mutagenized LacI binding site resulting in constitutive expression, Chlor^R^	This study
pRKJ-GFP	pBBR-derivative expressing GFP under control of Ptac, confers gentamicin resistance	This study

### Plasmid construction

Plasmid pXDC50 [9] was digested with *Hpa*I to remove the bulk of LacI and the N-terminal sequence of the mCherry coding sequence. A mutagenized promoter/operator library was constructed using Strand Overlap Extension (SOE) PCR. All oligonucleotide primer sequences are listed in supplemental materials (S1 Table). PCR fragments amplified from pXDC50 using primers MJG394 and MJG879 (containing degenerate bases in the *lacO*_*1*_ operator) were combined with a PCR fragment amplified using primers MJG880 and MJG36 to construct a *lacI-*Ptac*-mCherry construct (Ptac* indicates the degenerate operator binding sites within the library). The resulting fragments were digested with *Hpa*I, ligated into *Hpa*I-digested pXDC50, transformed by heat shock into chemically competent DH5α and plated onto LB plus chloramphenicol. The resulting transformants were carefully scraped from the plates and the mutagenized plasmid pool was purified using the Qiagen Mini Prep kit. The resulting plasmid library was then electroporated into *L*. *pneumophila* KS79 (see below). After recovering in AYE for 6 hours at 37°C, the transformation mixture was plated onto CYE agar plates supplemented with chloramphenicol and incubated for 3 days at 37°C. Clones expressing high levels of mCherry in *L*. *pneumophila* were purified to fresh CYE + chloramphenicol agar plates. The resulting plasmids were isolated and subjected to DNA sequencing. Plasmid pRKJ-GFP was constructed by amplifying the vector backbone of pXDC92 (kanamycin resistant, pBBR derivative) with primers MJG406 and MJG407. The resulting PCR product was ligated to a PCR fragment from pXDC31 (incompatibility group Q (IncQ) plasmid, GFP+ under control of P*tac*) containing the *lacI* gene and *gfp+* gene created with primers MJG394 and MJG36. The pON.mCherry plasmid is available from the AddGene plasmid repository and plasmid sequence information is available at the Figshare repository (https://doi.org/10.6084/m9.figshare.4667932.v1).

### Preparation of electrocompetent bacteria

*Legionella* strains were prepared for electroporation as follows. Freshly grown colonies were removed from CYE plates and suspended in 1 mL of ice cold 10% glycerol. The resulting suspension was centrifuged and washed 3 times with 1 mL of ice-cold 10% glycerol. Following the final wash, the cells were resuspended in 100 μL ice-cold 10% glycerol. Plasmid DNA (~1 μg) was mixed with the electrocompetent cells and incubated on ice for 1 minute prior to electroporation (2.4 kV, 100 Ω, 25 μF). One mL AYE broth was added and the cells were allowed to recover for 6 hours at 37°C prior to plating on the appropriate selective medium. For preparation of electrocompent *Vibrio cholerae*, colonies were spread onto a fresh LB plate and incubated for 4 hours at 37°C. Following this incubation, the cells were gently scraped off of the plate and suspended in 1 mL of ice cold 2 mM CaCl_2_. The suspension was centrifuged at 14,000 rpm in a tabletop micro centrifuge for 5 minutes at 4°C. The cell pellet was washed 2 additional times with 1 mL cold CaCl_2_ solution. The washed pellets were resuspended in fresh CaCl_2_ and plasmid DNA was introduced by electroporation (1.8 kV, 200 Ω, 25 μF) and allowed to recover in 1 mL of SOC medium [10] at 37°C for one hour prior to plating on selective medium. *Acinetobacter* and *Klebsiella* cultures were prepared for electroporation using overnight cultures. 1 mL aliquots were centrifuged at full speed in a tabletop micro-centrifuge for 2 minutes. The resulting cell pellets were washed 3 times with 2 volumes of room temperature 300 mM sucrose. Electrocompetent cells were electroporated and recovered as described above for the *Vibrio* cells.

### Fluorescence analysis of strains harboring pON.mCherry

Overnight cultures of bacteria were back-diluted 1:100 into fresh media containing the indicated amounts of IPTG. 100 μL volumes of the resulting dilutions were added to triplicate wells of a black-walled, clear bottomed 96 well microtiter plate (COSTAR, Corning, NY). Growth and mCherry fluorescence were monitored at 15-minute intervals in a Tecan M200 plate reader for 24 hours. Excitation/emission wavelengths for mCherry and GFP were 580/620 and 488/520 nm, respectively. Data are presented either as the entire growth curve or as a single point reading from the 16-hour time point. Growth curves were repeated three times and data from a single representative experiment are presented.

### Infection of amoebae

Bacterial infection of amoebae was performed as previously described [6]. Briefly, overnight cultures of bacteria were washed in AC buffer. Bacterial density was adjusted by optical density and added at a 1:1 bacteria:amoeba ratio to triplicate wells of a 96-well plate containing 1×10^5^ amoebae in AC buffer in a total of 100 μL. The 96 well plates were incubated at 30°C for *A*. *castellanii* and at 25°C for *D*. *discoideum* in a Tecan M200 microtiter plate reader and growth was measured by monitoring mCherry fluorescence over the indicated time as described above. In this assay, increases in mCherry fluorescence represent intracellular bacterial growth.

For determination of bacterial growth in *A*. *castellanii* by measuring colony forming units (CFU), the infections were initiated as described above with the exception that the infections were conducted in a total volume of 200 μL. 10μL aliquots of each well were serially diluted and plated onto CYE to determine the total CFU/well at the indicated time points. CFU recovery assays were repeated in triplicate and results from a representative experiment are shown.

## Results

### Construction of pON.mCherry

**Fig 1:**
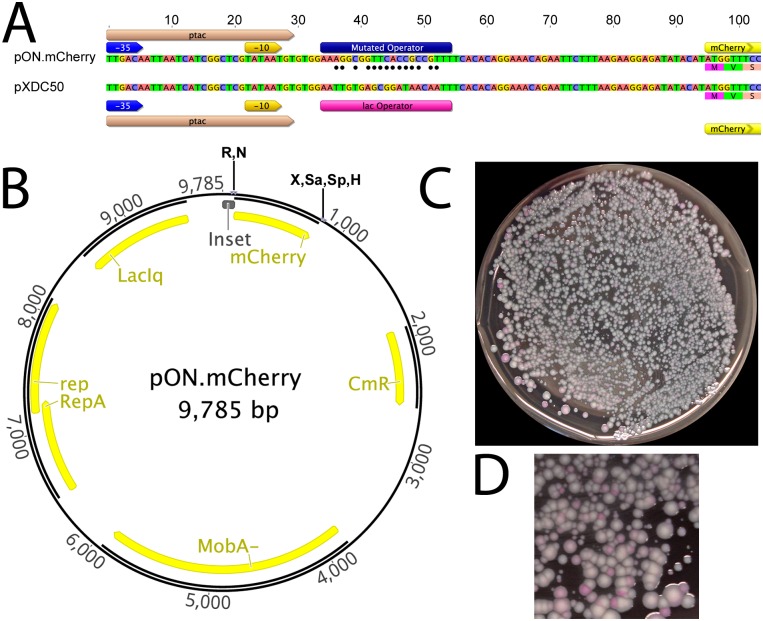
Construction of pON.mCherry. **(A)** Promoter regions of pXDC50 (parental plasmid) and pON.mCherry. DNA fragments from a mutagenic PCR strategy targeting the lac repressor-binding site were cloned into pXDC50 as described in the Materials and Methods. Black circles identify nucleotide changes between the parental pXDC50 *lacO1* operator and the mutated operator in pON.mCherry. **(B)** pON.mCherry plasmid map. Unique restriction sites are shown; R, EcoRI; N, NdeI; X, XbaI; Sa, SalI; Sp, SphI; H, HindIII. **(C), (D)** Screening pON.mCherry library in *L*. *pneumophila* demonstrates a variety of promoter strengths in the pON mutant operator library.

**Fig 2:**
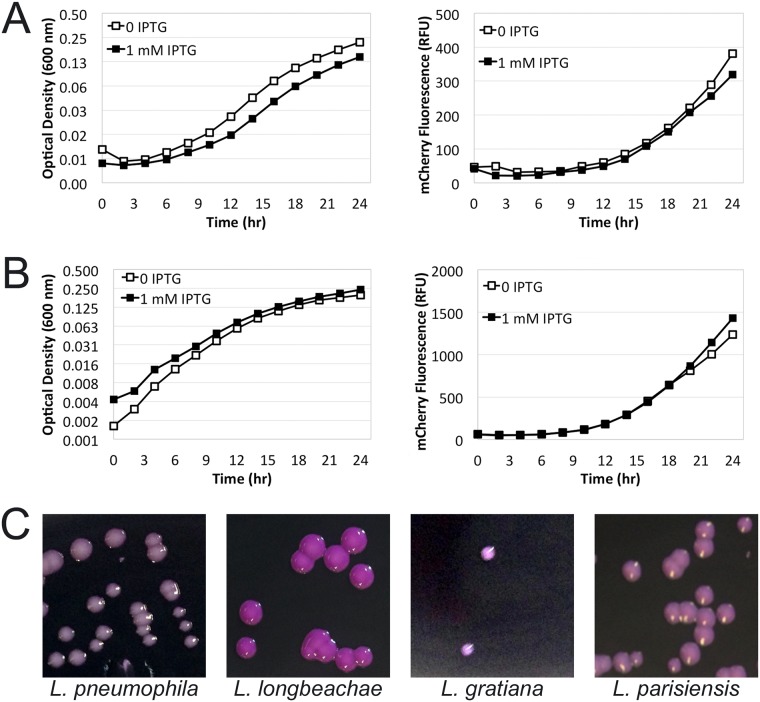
pON.mCherry yields constitutive mCherry expression in several *Legionella* species. **(A) & (B)** Growth curves showing mCherry expression in (A) *L*. *pneumophila* and (B) *L*. *longbeachae*. Strains harboring the indicated plasmids were monitored for growth (left panels; log_2_ scale) and mCherry expression (right panels; linear scale) in the presence and absence of 1 mM IPTG. **(C)** Photographs of mCherry expression in the indicated *Legionella* species.

In order to develop a plasmid that constitutively expresses a target gene, we chose to use a broad-host range plasmid, pXDC50. A derivative of the incompatibility group Q (IncQ) plasmid pMMB207C [6], pXDC50 harbors the coding sequence of mCherry, a red fluorescent protein, under control of the IPTG-inducible P*tac* promoter [9]. We used an overlap extension PCR method with an oligonucleotide primer containing random bases within the *lac* operon repressor (LacI) binding site (*lacO*_*1*_ operator) of pXDC50 (Fig 1, Materials and methods). These mutagenized promoter regions were cloned into the *lac* promoter region of pXDC50 and transformed into DH5α. Because the plasmid was originally intended for use in *Legionella pneumophila*, we extracted plasmid DNA from the library of DH5α transformants and introduced the resulting plasmid pool into *L*. *pneumophila* in order to identify clones that express high levels of mCherry in the absence of IPTG specifically in *L*. *pneumophila*. A representative plate of the transformation mixture is shown in Fig 1C and 1D. A total of eight clones expressing high levels of mCherry were selected for further analysis, and of these, a single clone was subjected to DNA sequencing of the *lacO*_*1*_ operator region. The resulting plasmid was given the name pON.mCherry. Interestingly, the level of mCherry production appeared variable when pON.mCherry was introduced to other *Legionella* species (Fig 2).

Sequence analysis of the promoter region of pON.mCherry reveals significant disruption of the *lac* operator of pON.mCherry (Fig 1A). The parental plasmid, pXDC50, contains the *tac* promoter sequence and the *lacO*_*1*_ operator (5’-AATTGTGAGCGGATAACAATT-3’) [7]. As shown in Fig 1, the *lac* operator region on pON.mCherry has mutations in 14 of the 21 nucleotides that comprise the *lacO*_*1*_ operator sequence; the operator sequence in pON.mCherry is 5’-AA*AG*G*C*G*GTTCACCGC*CC*GT*T-3’ (underlined, italic text indicates nucleotides altered from the WT *lacO*_*1*_ sequence). Comparing the mutated operator sequence to previous structural studies provides an explanation for the lack of repression observed with pON.mCherry. Two key specificity-determining residues of LacI are Leucine-6 (Leu^6^) and Arginine-22 (Arg^22^) [11, 12]. In the structure of LacI bound to the *lacO1* operator, Leu^6^ forms hydrophobic contacts with nucleotides Thymidine-8 and Cytosine-9 on the complementary strand of *lacO1* and in the pON.mCherry operator site, these nucleotides are mutated to a Cytosine and an Adenine, respectively. Arg^22^ forms hydrophobic interactions with Thymine nucleotides 4 and 6 in the major groove of the WT *lacO*_*1*_ operator sequence. In the pON.mCherry operator sequence, these bases have been mutated to a Guanine at position 4 and a Cytosine at position 6, possibly eliminating these hydrophobic interactions. While Arg^22^ also participates in hydrogen bonding with Guanine-5, and this nucleotide remains unchanged in the pON.mCherry, this interaction itself is likely insufficient to mediate repressor binding.

### pON.mCherry does not interfere with IPTG induction of other genes

**Fig 3:**
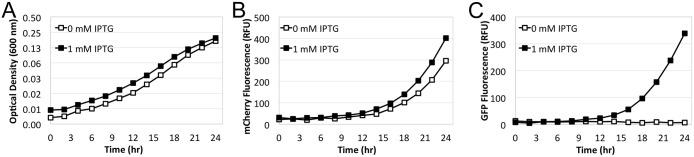
pON.mCherry does not interfere with induction with other P*tac* driven plasmids. *L*. *pneumophila* KS79 harboring both pON.mCherry and pRKJ-GFP was cultured in a 96 well micro-titer plate and growth (A), mCherry production (B) and GFP production (C) were monitored over time in the presence and absence of 1 mM IPTG. As in Fig 2, increases in optical density are plotted on a log_2_ scale.

Because pON.mCherry harbors an intact *lacI* gene, encoding the *lac* repressor, we first sought to determine if the presence of pON.mCherry would interfere with other plasmids that rely on P*tac*/IPTG for expression. Towards this end, we constructed pRKJ-GFP, a pBBR derivative with GFP expression under control of P*tac*. As can be seen in Fig 3, *L*. *pneumophila* harboring both pRKJ-GFP and pON.mCherry produced similar levels of mCherry fluorescence independently of IPTG, while GFP expression was only observed upon addition of IPTG to the medium.

### pON.mCherry expression does not interfere with the ability of *Legionella* to cause infection

**Fig 4:**
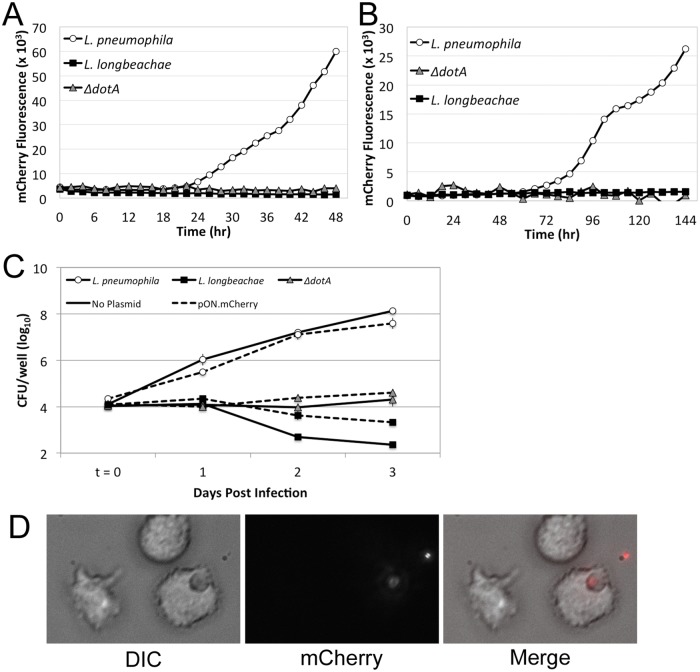
Infection of amoebae with *Legionella* strains harboring pON.mCherry. *A*. *castellannii* **(A)** and *D*. *discoideum* **(B)** were infected with the indicated strains of bacteria harboring pON.mCherry and incubated at 30°C or 25°C, respectively, for the indicated times. **(C)** Bacterial growth was monitored by colony forming units recovered from the infections of *A*. *castelannii* at the indicated days post infection for strains without a plasmid (solid lines) and also harboring pON.mCherry (dashed lines). **(D)** Representative micrographs of *A*. *castellanii* cells infected with *L*. *pneumophila* harboring pON.mCherry. Left panel, phase contrast image; Right panel, mCherry image. All infections were performed at a multiplicity of infection (MOI) of 1 and repeated in triplicate. Results from a representative experiment are shown.

*L*. *pneumophila* is an intracellular pathogen and the causative agent of Legionnaires’ Disease (LD) [13]. In the United States, *L*. *pneumophila* is identified in the majority of cases of LD, although, increasing proportions of LD are now being attributed to *L*. *longbeachae* infection, which has historically been found more frequently in the southern hemisphere [14, 15]. Outside of the human host, *Legionella* are thought to inhabit a variety of free-living amoebal hosts [16]. A common approach for investigating the host-pathogen interactions of *Legionella* and ameobae utilizes mCherry expression as a proxy for bacterial growth in the amoeba [6]. As such, we wished to assess the utility of pON.mCherry as a tool for monitoring *Legionella* growth within amoebae. Both *L*. *pneumophila* and *L*. *longbeachae* harboring pON.mCherry were used to infect the ameobal species *Acanthamoeba castellanii* and *Dictyostelium discoideum*. It has been previously demonstrated that wild type *L*. *pneumophila* readily infect and replicate within *A*. *castellanii* whilst *L*. *longbeachae* does not [17]. As a control, an *L*. *pneumophila* strain harboring a deletion of a critical Type IVb secretion system gene, *dotA*, was included in these studies and failed to grow. Importantly, we did not observe significant differences in bacterial growth when measured by colony forming units from isogenic strains lacking the pON.mCherry plasmid (Fig 4). We were also able to visualize *L*, *pneumophila* uptake by *A*. *castellanii* cells, suggesting that pON.mCherry can be used for fluorescent microscopy studies as well (Fig 4D). Taken together, these data show that pON.mCherry does not prevent the growth of the bacteria or their ability to interact with either amoebal species.

### pON.mCherry functions in a variety of Gram-negative bacterial species

**Fig 5:**
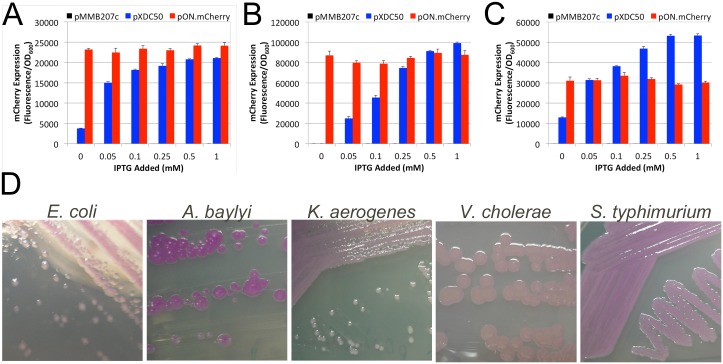
pON.mCherry functions in several Gram-negative bacterial species. Plasmids (pMMB207c, black bars; pXDC50, blue bars; pON.mCherry, red bars) were introduced into several Gram-negative bacteria, including **(A)** *E*. *coli*, **(B)** *Acinetobacter baylyi* ADP1 and **(C)** *Salmonella typhimurium* LT2. The resulting strains were grown for 16 hours in the presence of increasing concentrations of IPTG. mCherry expression is displayed as the mCherry fluorescence divided by optical density to normalize expression values to bacterial growth with error bars representing one standard deviation across three triplicate wells. The experiment was performed in triplicate and data from a representative experiment is shown. **(D)** Representative photographs showing colonies of the indicated strains harboring pON.mCherry after overnight growth on LB plates at 37°C.

As the *lac* promoter is functional in a variety of bacterial species [18] and the pON.mCherry replicon backbone (pMMB207C; IncQ) is known to replicate in a wide array of bacterial species [19], we wished to ascertain if pON.mCherry also functions in a diverse collection of Gram-negative bacterial species, including several *Legionella* species (Fig 2C), *Acinetobacter baylyi*, *Escherichia coli*, *Klebsiella aerogenes*, *Salmonella typhi* and *Vibrio cholorae*. As shown in Fig 5, several species of bacteria harboring pON.mCherry produce mCherry protein. Furthermore, comparing mCherry fluorescence in strains harboring either pON.mCherry or the parental plasmid, pXDC50, demonstrates that pON.mCherry neither requires nor responds to the presence of IPTG in the growth medium for expression of mCherry (Fig 5). Interestingly, as observed when pON.mCherry is introduced into other *Legionella* species (Fig 2C), the amounts of mCherry produced varied in the different Gram-negative species (Fig 5).

## Discussion

Previous attempts in our lab to generate a constitutive *lac* promoter entailed generating single point mutations within the *lac* operator that are known to lead to constitutive *lac* expression in *E*. *coli* [20], but the resulting plasmids still responded to IPTG in *Legionella* (data not shown). While other researchers have developed constitutive expression systems in *Legionella pneumophila*, the pON.mCherry plasmid described herein provides several advantages. For example, Tiaden and colleagues generated a similar plasmid, pNT28, which constitutively drives *gfp* expression [21]. The constitutive expression from pNT28 plasmid derives from disruption of the *lacI* gene on the plasmid backbone but the *lac* operator is intact. As such, expression from pNT-28 would again require IPTG in the presence of a functional Lac Repressor supplied from a second compatible vector or if the bacterial host encodes such a repressor.

As a result of these shortcomings, we employed a polymerase chain reaction (PCR) based method in conjunction with a degenerate oligonucleotide primer to randomly mutagenize the lac operator of plasmid pXDC50 *in vitro*. This allowed us to screen a library of operator mutants *en masse* by transforming the resulting plasmid pool directly into *Legionella*. An extension of this approach would allow one to develop constitutive promoters in any bacterial strain that can initiate transcription from the strong P*tac* promoter [7, 18]. Importantly, while the commonly used P*tac* promoter mediates the expression from pON.mCherry, the presence of pON.mCherry does not interfere with other plasmids in the same cell that utilize the P*tac* system for induction (Fig 3). This allows for constitutive expression of mCherry and the controlled expression of another gene of interest from a compatible vector. Furthermore, we observed that the expression of pON.mCherry is not detrimental to the growth/interaction of *L*. *pneumophila* within amoeba hosts (Fig 4) and the resulting plasmid construct also functions in a variety of other Gram-negative species (Figs 2 and 5). One interesting observation, as mentioned above and shown in Figs 2 and 5, is that the level of mCherry expression varies across the different bacterial species. We speculate that these differences could result from several factors. One potential explanation is that P*tac* promoter activity differs across the tested species. Additional possibilities leading to differential mCherry expression include differences in the mCherry mRNA lifetime; translation efficiency of the mRNA; and/or the stability/turnover rate of the mCherry protein.

Herein, we describe the construction of a broad-host range plasmid that constitutively expresses mCherry in a wide range of bacterial species. The resulting plasmid, pON.mCherry, takes advantage of the strong P*tac* promoter, which has been shown to function in many bacterial species, and eliminates the need for adding an inducing substance for protein expression. In addition pON.mCherry harbors several useful restriction endonuclease recognition sites (Fig 1B), facilitating the cloning of other genes into this plasmid. Using the pON backbone we have already employed this approach for several genes of interest in our own laboratory for constitutive expression in *L*. *pneumophila* and other *Legionella* species. Furthermore, the approach employed to generate pON.mCherry, namely performing degenerate PCR of a region harboring the binding site of a regulatory protein, could be applied to other expression systems and/or organisms.

## Supporting information

S1 Table Oligonucleotide primers used in this study. (PDF)
