# Supplementary material for: Seeing red; the development of pON.mCherry, a broad-host range constitutive expression plasmid for Gram-negative bacteria
Source: PLoS One. 2017 Mar 3;12(3):e0173116. doi: 10.1371/journal.pone.0173116 (PMC5336243; doi:10.1371/journal.pone.0173116)
Supplement: S1 Table — (PDF) [file pone.0173116.s001.pdf]

Table S1. Oligonucleotide Primers used in this study

Primer	Sequence (5’-3’)
MJG036	CAGACCGCTTCTGCGTTCTGAT
MJG394	TAGGCCGCTTTCCTGGCTTTG
MJG406	CCAAGCGGCCGGAGAACCTG
MJG407	GCCTTGCAGCACATCCCCCT
MJG879	GAATTCTGTTTCCTGTGTGAAANNNNNNNNNNNNNNNNNTTCCACACATTATACGAGCC
MJG880	TTTCACACAGGAAACAGAATTCTTTAAGAAGG
